# Seizure Semiology, EEG, and Imaging Findings in Epilepsy Secondary to Mitochondrial Disease

**DOI:** 10.3389/fneur.2021.779052

**Published:** 2021-11-29

**Authors:** Anthony L. Fine, Greta Liebo, Ralitza H. Gavrilova, Jeffrey W. Britton

**Affiliations:** ^1^Department of Neurology, Mayo Clinic, Rochester, MN, United States; ^2^Department of Radiology, Mayo Clinic, Rochester, MN, United States; ^3^Department of Clinical Genomics, Mayo Clinic, Rochester, MN, United States

**Keywords:** EEG, mitochondria, genetic, neuroimaging, epilepsy

## Abstract

**Background:** Identification of an underlying mitochondrial disorder can be challenging due to the significant phenotypic variability between and within specific disorders. Epilepsy can be a presenting symptom with several mitochondrial disorders. In this study, we evaluated clinical, electrophysiologic, and imaging features in patients with epilepsy and mitochondrial disorders to identify common features, which could aid in earlier identification of a mitochondrial etiology.

**Methods:** This is a retrospective case series from January 2011 to December 2019 at a tertiary referral center of patients with epilepsy and a genetically confirmed diagnosis of a mitochondrial disorder. A total of 164 patients were reviewed with 20 patients fulfilling inclusion criteria.

**Results:** A total of 20 patients (14 females, 6 males) aged 0.5–61 years with epilepsy and genetically confirmed mitochondrial disorders were identified. Status epilepticus occurred in 15 patients, with focal status epilepticus in 13 patients, including 9 patients with visual features. Abnormalities over the posterior cerebral regions were seen in 66% of ictal recordings and 44% of imaging studies. All the patients were on nutraceutical supplementation with no significant change in disease progression seen. At last follow-up, eight patients were deceased and the remainder had moderate-to-severe disability.

**Discussion:** In this series of patients with epilepsy and mitochondrial disorders, we found increased propensity for seizures arising from the posterior cerebral regions. Over time, electroencephalogram (EEG) and imaging abnormalities increasingly occurred over the posterior cerebral regions. Focal seizures and focal status epilepticus with visual symptoms were common. Additional study is needed on nutraceutical supplementation in mitochondrial disorders.

## Introduction

Disorders of mitochondrial function and metabolism result in energy failure producing varying severity of symptoms (1–3). The central nervous system is particularly susceptible to mitochondrial defects and neurologic manifestations are frequently seen in mitochondrial disorders (4, 5).

Seizures are particularly common in mitochondrial disorders such as polymerase gamma (POLG) mutations, mitochondrial encephalomyopathy, lactic acidosis, and stroke-like episodes (MELAS), and myoclonic epilepsy with ragged-red fibers (MERRF) (6–9). Published information on specific seizure semiology and electroencephalogram (EEG) findings in mitochondrial disorders is limited compared to what is known about other genetic and metabolic epilepsies, likely owing to the significant variability between and within specific mitochondrial disorders (10). While seizures are not unique to a mitochondrial etiology (11–13), it has been previously noted that EEG abnormalities in these disorders tend to have a posterior cerebral predilection (14–16). Within the central nervous system, mitochondrial dysfunction frequently results in structural injury, which can manifest as characteristic neuroimaging abnormalities (17, 18). The time to diagnosis can be variable and in some cases may take years when presentations are atypical or when symptoms and diagnostic studies are non-specific. Treatment of these disorders is limited and mainly comprised of utilization of nutritional therapies aimed to increase the metabolic substrates used in mitochondrial processes (19). These nutraceutical therapies, sometimes referred to as the “mitochondrial cocktail,” are unproven at present with limited evidence-based support of their use.

The main objective of this study was to evaluate the EEG, clinical, and radiologic progression of disease in patients with epilepsy due to mitochondrial cytopathies in order to identify features, which may help the clinician more readily diagnose these disorders. A secondary objective was to study nutraceutical therapy use in relationship to disease progression.

## Materials and Methods

This retrospective study was performed using the Mayo Clinic Mitochondrial Disease Database from January 2011 to December 2019. Records were reviewed for diagnosis, history of epilepsy, laboratory investigations, and available raw EEG and MRI studies. Patients were classified based on previously published diagnostic criteria as having a definite, probable, or possible mitochondrial disorder (20–23).

Patients with a definite, genetically confirmed diagnosis of mitochondrial disorder were included. Patients without confirmatory testing or where there was diagnostic ambiguity were excluded. Additional inclusion criteria included history of seizures and EEG and MRI studies performed at our institution. Patients were excluded if any of the inclusion criteria were missing or if medical records were limited.

Information extracted from the medical record included seizure history and semiology, anti-seizure therapies and side effects, laboratory investigations, including genetic testing and tissue biopsy results, EEG and neuroimaging findings, use of nutraceuticals/supplements, and disposition at last follow-up. Seizure semiology was classified based upon the most recent International League Against Epilepsy (ILAE) classification schema (24, 25).

Electroencephalogram studies were graded based on the degree and location of abnormalities and rated as either normal or demonstrating mild, moderate, or severe abnormalities using the Mayo Clinic EEG classification system, which assigns a dysrhythmia score of 1 through 3 (1 = mild, 2 = moderate, and 3 = severe). This included pediatric EEG recordings, which were reviewed and interpreted with attention to age-expected findings in children. “Mild” was defined as background slowing of 7–8 Hertz (Hz) or intermittent slowing in the theta range (5–7 Hz); “moderate” if there was a mixture of frequent delta-theta slowing (<4 Hz up to 7 Hz) or if there was a mild-to-moderate asymmetry of the background (<50% difference in amplitude between homologous regions); and “severe” if any of the following were present: focal or diffuse delta slowing (<4 Hz), severe background asymmetry (>75% difference in amplitude between homologous regions), epileptiform abnormalities [spikes, spike and wave, and sharp wave discharges, lateralized periodic discharges (LPDs) or generalized periodic discharges (GPDs), and periodic sharp wave complexes], or recorded seizures or status epilepticus. The locations of EEG abnormalities were noted and categorized as focal, multifocal (focal abnormalities in more than two regions), or generalized.

Magnetic resonance imaging studies were reviewed by one study author (GL) and abnormalities noted based on location, imaging sequence [T2/fluid-attenuated inversion recovery (FLAIR) and diffusion-weighted imaging (DWI)], abnormal contrast enhancement, and presence or absence of cortical laminar necrosis, atrophy, or encephalomalacia.

Electroencephalogram and MRI results were evaluated for anatomic concordance, defined as colocalization on studies, comparing studies obtained within 30 days of each other. Serial EEG and MRI studies were also evaluated for disease progression, defined as the presence of new abnormalities compared to previous studies.

Functional status was assigned based on initial and last documented encounters, using the modified Rankin scale (mRS), with a score of 0 (no symptoms), 1 (no significant disability), 2 (slight disability), 3 (moderate disability), 4 (moderate-to-severe disability), 5 (severe disability), or 6 (death). Patient disposition was determined at last known follow-up based on notes or communications contained in the medical record.

This study was approved by the Mayo Clinic Foundation Institutional Review Board.

## Results

A total of 164 unique patient records were reviewed with 144 patients excluded for the following reasons: limited/missing clinical information (*n* = 22), absence of seizures (*n* = 113), or diagnosis of a probable or possible mitochondrial disorder (*n* = 9).

Diagnoses in excluded patients included MELAS (m.3243A>G variant) (*n* = 28), chronic progressive external ophthalmoplegia (CPEO)/Kearns–Sayre syndrome (KSS) (*n* = 14), Leber hereditary optic neuropathy (LHON) (*n* = 5), neuropathy, ataxia, and retinitis pigmentosa (NARP) (*n* = 2), and Leigh syndrome (*n* = 2). In an additional 34 patients without seizure, the diagnosis was based on history and laboratory findings without genetic confirmation.

Table 1 shows details on the 20 patients that fulfilled inclusion criteria. The mean age at initial evaluation was 23 years (median 21.5 years, SD 14.3 years) with 6 males and 14 females. Two kindred pairs were included (one sibling pair and one parent–child pair). The most common diagnoses were MELAS (*n* = 12) and POLG1 (*n* = 3). Evaluation of epilepsy was the most common initial visit indication (*n* = 14), followed by genetics evaluation (*n* = 2), developmental delay (*n* = 2), and headache evaluation (*n* = 2).

**Table 1:** Seizure characteristics and functional status of patient.

**Patient**	Age^*^, Sex	**Diagnosis**	**Semiology**	**Status epilepticus**	**EEG findings**	**Antiseizure medications used**	**Medication side effects**	**Initial modified Rankin scale**	**Last modified Rankin scale**
1	22 y, F	POLG-related epilepsy	Focal motor seizures. Myoclonic seizures.	Focal motor status/EPC. Myoclonic status.	Right posterior LPDs	VPA, PB, LEV, LCS, CZP, PHT	Hyperammonemia	2	6 (dec. 22 y)
2	32 y, F	MELAS	Focal impaired awareness seizures	N/A	Normal	N/A	N/A	3	4
3	6 mo, F	Leigh syndrome	Epileptic spasms	N/A	Multifocal independent spikes. GASW. Hypsarrhythmia pattern	VIG, TPM	None	4	6 (dec. 12 mo)
4	23 y, F	MERFF	Focal aware sensory (visual) seizures. Myoclonic seizures	Focal sensory (visual) status	Occipital LPDs.	LEV, PHT, LCS, LTG, TPM	Transaminitis, dizziness, nausea	0	3
5	30 y, F	MELAS	Focal impaired awareness non-motor seizures	Focal non-motor status	Left temporal LPDs and occipital spikes	VPA, LEV, LTG, PB, LCS, GBP	Rash, depression	1	4
6	30 y, M	SANDO/POLG	Focal impaired awareness seizures. Focal to bilateral tonic-clonic seizures	N/A	Excess generalized beta activity	VPA, OXC	Transaminitis	1	5
7	18 y, F	SANDO/POLG	Focal sensory (visual) impaired awareness seizures. Myoclonic seizures	Focal motor status/EPC	Multifocal independent spikes and sharp waves. Left occipital LPDs	PHT, LEV, LCS, OXC, TPM, CZP, VPA	Transaminitis, ataxia, sedation, diplopia	1	4
8	40 y, M	MELAS	Focal impaired awareness sensory (visual) seizures	Focal non-motor sensory (visual) status	Mild diffuse nonspecific slowing	LEV, PHT	Insomnia	0	6 (dec. 51 y)
9	17 y, F	MELAS	Focal to bilateral tonic-clonic seizures	N/A	Moderate diffuse non-specific slowing	LTG, OXC, CZP	None	2	2
10	20 y, F	MELAS	Focal impaired awareness sensory (visual) seizures	Focal non-motor sensory (visual) status	Moderate focal temporo-occipital slowing	PHT, LEV, LTG	Mood change	0	5
11	47 y, F	MELAS	Focal to bilateral tonic-clonic seizures	Generalized tonic-clonic status	Multifocal LPDs, severe focal parieto-occipital slowing	PHT, LEV	None	1	6 (dec. 57 y)
12	21 y, F	MELAS	Focal to bilateral tonic-clonic seizures	Focal non-motor sensory (visual) status	Occipital spikes. Posterior predominant GASW	LEV, PGB, GBP, LTG, PHT, ESM	Headache	0	6 (dec. 23 y)
13	16 y, M	SANDO/POLG	Focal aware sensory (visual) seizures. Focal to bilateral tonic-clonic seizures	N/A	GASW	PHT, CBZ	None	0	5
14	22 y, F	MELAS	Focal impaired awareness sensory (visual) seizures	Focal sensory (visual) status	Bilateral occipital spikes and sharp waves. GASW	LEV, GBP	None	0	3
15	22 y, F	MELAS	Focal aware sensory (visual) seizures	Focal motor status/EPC	Excess generalized beta activity	LEV	None	0	2
16	3 y, F	Ponto-cerebellar hypoplasia type 1B	Focal to bilateral tonic-clonic seizures	Generalized tonic-clonic status	FIRDA. Independent bilateral centroparietal LPDs	None	N/A	3	3
17	12 y, F	DNM1L-related epileptic encephalopathy	Generalized tonic-clonic seizures. Focal motor seizures. Myoclonic seizures	Generalized tonic-clonic status. Focal motor status. Myoclonic status.	GPDs. Focal right central slowing and electrographic seizures	PB, LEV, LCS, FLB, CZP, ketogenic diet	Renal failure	1	6 (dec. 13 y)
18	13 y, M	MELAS	Focal motor seizures	Generalized tonic-clonic status. Focal sensory (visual) status	Right occipital LPDs. Posterior predominant multifocal spikes	LEV, PHT, LTG, CZP	None	0	6 (dec. 29 y)
19	12 y, M	MELAS	Focal aware sensory (visual) seizures	Focal sensory (visual) status	Bi-parieto-occipital LPDs	VPA, LEV, LCS, LTG, PHT	Stevens-Johnson syndrome	0	6 (dec. 25 y)
20	61 y, M	MELAS	Focal impaired awareness seizures	Focal non-motor status	Independent bitemporal LPDs	LEV	None	1	5

* *Age at presentation*.

*dec, deceased; DNM1L, dynamin-like protein 1; EPC, epilepsia partialis continua; ESM, ethosuximide; FB, felbamate; GBP, gabapentin; GASW, generalized atypical spike and wave; LCS, lacosamide; LEV, levetiracetam; LPDs, lateralized periodic discharges; LTG, lamotrigine; MELAS, mitochondrial encephalomyopathy, lactic acidosis, and stroke-like episodes; MERFF: myoclonic epilepsy with ragged-red fibers; OXC, oxcarbazepine; PB, phenobarbital; PGB, pregabalin; PHT, phenytoin; POLG, polymerase gamma; SANDO, sensory ataxia, neuropathy, dysarthria, and ophthalmoparesis; TPM, topiramate; VIG, vigabatrin; VPA, valproic acid*.

### Metabolic and Genetic Testing

All the patients underwent biochemical and metabolic analyses including some combination of blood, urine, and cerebrospinal fluid (CSF) testing (Table 2). Studies commonly performed included urine organic acids, serum amino acids, serum lactate and pyruvate, comprehensive fatty acid profile, carnitine, and acylcarnitine profile. The most commonly seen abnormalities on blood testing were an elevated lactate (*n* = 17), pyruvate (*n* = 10), and alanine (*n* = 7). Spinal fluid analysis was performed in seven patients, with elevated lactate and pyruvate seen in two patients and elevated CSF protein (>35 mg/dl) in six patients. Muscle biopsy was performed in five patients and fibroblast culture and analysis was performed in three patients (Table 2).

**Table 2:** Phenotypes, genetic, and biochemical testing of patient.

**Patient**	**Age, sex**	**Phenotype**	**Diagnosis**	**Gene, variant**	**Heteroplasmy (source)**	**Biochemical testing**	**Muscle biopsy /fibroblasts**
1	22 y, F	Developmental delay, epilepsy	POLG-related epilepsy	POLG1, c.1491G>C, POLG1, c.2243G>C	N/A	Elevated serum lactate	Cytochrome c oxidase negative fibers
2	32 y, F	Short stature, thin body habitus, hearing loss, epilepsy, diabetes, cardiac arrhythmias, ataxia	MELAS	MTTL1, m.3243A>G	Unknown	Elevated serum lactate, pyruvate, and alanine	N/A
3	6 mo, F	Developmental delay, failure to thrive, infantile spasms, hypotonia, Wolf-Parkinson White syndrome	Leigh syndrome	ND5, m.13513G>A	72.6% (blood)	Elevated serum lactate and pyruvate	N/A
4	23 y, F	Short stature, thin body habitus, epilepsy, intellectual disability	MERFF	MTTL1, m.3256C>T	40.7% (blood)	Elevated serum lactate	Fibroblasts: normal.
5	30 y, F	Stroke-like episodes, focal epilepsy, migraine, peripheral neuropathy	MELAS	ND5, m.13513G>A	10% (blood) 77% (muscle)	Elevated serum lactate, pyruvate, and alanine	N/A
6	30 y, M	Progressive external ophthalmoplegia, epilepsy, developmental delay/intellectual disability, mood disorder, peripheral neuropathy, ataxia	SANDO	POLG1, c.1491G>C, POLG1, c.2243G>C	N/A	Normal	Increased sarcolemmal oxidative reactivity
7	18 y, F	Progressive external ophthalmoplegia, epilepsy, mood disorder, peripheral neuropathy, ataxia	SANDO	POLG1, c.1491G>C, POLG1, c.2243G>C	N/A	Normal	N/A
8	40 y, M	Short stature, hearing loss, diabetes, cardiomyopathy, stroke-like episodes, epilepsy	MELAS	MTTL1, m.3243A < G	Unknown	Elevated serum lactate and pyruvate	N/A
9	17 y, F	Global developmental delay, spasticity, intellectual disability, progressive external ophthalmoplegia, epilepsy	MELAS	MTTL1, m.3243 A>G	73% (blood)	Elevated serum lactate and alanine	N/A
10	20 y, F	Thin body habitus, failure to gain weight, Stroke-like episodes, hearing loss, epilepsy, cardiac arrhythmia	MELAS	MTTL1, m.3243A>G	59% (blood)	Elevated serum lactate, pyruvate, and alanine	N/A
11	47 y, F	Stroke-like episodes, hearing loss, diabetes, short stature, epilepsy	MELAS	MTTL1, m.3243A>G	97% (blood)	Elevated serum lactate	Focal subsarcolemmal fuchsinophilic material on trichrome stain
12	21 y, F	Epilepsy, peripheral neuropathy, myopathy, stroke-like episodes	MELAS	MTTL1, m.3243 A>G	89.6% (muscle)	Elevated serum lactate and pyruvate	N/A
13	16 y, M	Migraines, peripheral neuropathy, ataxia, dysarthria, progressive external opthalmoplegia	SANDO	POLG1, c.1399G>A	N/A	Normal	N/A
14	22 y, F	Stroke-like episodes, epilepsy, ataxia, diabetes, hearing loss, cardiomyopathy	MELAS	MTTL1, m.3243A>G	Unknown	Elevated serum lactate	N/A
15	22 y, F	Short stature, thin body habitus, progressive external opthalmoplegia, ataxia, myopathy, cardiomyopathy, myoclonus, epilepsy	MELAS	MTTL1, m.3251A>G	92% (muscle)	Elevated serum lactate, pyruvate, and alanine	Ragged red fibers with cytochrome c oxidase activity
16	3 y, F	Global developmental delay, hypotonia, spasticity, epilepsy, ataxia	Ponto-cerebellar hypoplasia type 1B	EXOSC3, c.395A>C (homozygous)	N/A	Elevated serum lactate	Fibroblasts: abnormal mitochondrial cristae arrangements
17	12 y, M	Global developmental delay, speech apraxia, dystonia	DNM1L-related epileptic encephalopathy	DNM1L, c.1207C>T	N/A	Elevated serum lactate and pyruvate	Fibroblasts: increased lipid storage.
18	13 y, M	Stroke-like episodes, epilepsy, headaches, hearing loss	MELAS	MTTL1, m.3243A>G	70% (muscle)	Elevated serum lactate and pyruvate	N/A
19	12 y, M	Stroke-like episodes, epilepsy, headaches, mood disorder	MELAS	MTTL1, m.3243A>G	40% (blood)	Elevated serum lactate and alanine	Ragged blue fibers, increased succinate dehydrogenase enzyme reactivity
20	61 y, F	Ataxia, hearing loss, diabetes, stroke-like episodes, migraines	MELAS	MTTL1, c.3243A>G	6% (blood)	Elevated serum and CSF lactate, pyruvate, and alanine	N/A

*CSF, cerebrospinal fluid; DNM1L, dynamin-1-like protein; MELAS, mitochondrial encephalomyelopathy, lactic acidosis, and stroke-like episodes; MERFF, myoclonic epilepsy with ragged-red fibers; POLG, polymerase gamma; SANDO, sensory ataxia, neuropathy, dysarthria, and ophthalmoparesis*.

Genetic testing performed included point mutation analysis (*n* = 9), next-generation sequencing (*n* = 8), and whole-exome sequencing (*n* = 3). Heteroplasmy analysis was available in 11 patients with values ranging from 6 to 97% (Table 2). In the patient with 6% heteroplasmy in blood, the clinical phenotype was felt to be consistent with MELAS, with m.3243A>G variant, and muscle biopsy was recommended but declined by the patient.

### Seizure Semiology

The most common seizure semiology was focal seizures in 19 patients, with the exception of the patient with epileptic spasms. Focal semiologies included focal impaired awareness (*n* = 8), focal aware sensory seizures (*n* = 4), focal to bilateral tonic-clonic seizures (*n* = 6), and focal motor seizures (*n* = 3). This includes two patients who had both focal and focal to bilateral tonic-clonic seizures and one patient with a history of both focal motor seizures and generalized tonic-clonic seizures (although focal to bilateral tonic-clonic seizures cannot be excluded). Ictal visual sensory symptoms were experienced by eight patients (40%), with four patients who had retained awareness and four patients who experienced loss of awareness during seizures. Visual features described included ictal blindness, visual distortions, scintillating scotomas, hallucinations, palinopsia, and photopsia.

A total of 15 patients (75%) had a history of status epilepticus including 4 patients as their initial presenting symptom. Focal status epilepticus occurred in 13 patients, with focal motor status/epilepsia partialis continua occurring in 4 patients and focal non-convulsive status seen in 9 patients, with 7 patients experiencing focal sensory status with visual symptoms. Four patients had generalized tonic-clonic status epilepticus including two patients who experienced both focal and generalized status epilepticus on different occasions. Two patients experienced super-refractory myoclonic status epilepticus, with ultimate withdrawal of medical support resulting in death.

### Electroencephalogram Findings

A total of 64 EEG recordings were performed: 37 outpatient routine recordings and 27 inpatient continuous recordings. A total of 14 patients had more than one EEG recording performed (range 2–11, mode 2). Eight patients had both outpatient routine and inpatient continuous recordings performed. In the remaining patients, an additional eight patients had only outpatient routine recordings and four patients had only prolonged hospital studies performed.

Outpatient EEG studies were classified as normal in two patients, mildly abnormal in three patients (excess fast activity or excessive drowsiness), moderately abnormal in three patients (focal delta-theta slowing), and severely abnormal in eight patients [generalized atypical spike and wave (four patients), focal and multifocal discharges (three patients), and lateralized periodic discharges (one patient)]. Two of the four patients with generalized atypical spike and wave activity had additional focal epileptiform findings and ictal EEG recordings contained focal onset seizures.

Prolonged inpatient recordings were performed in 12 patients (60%). All prolonged recordings were classified as severely abnormal. Epileptiform abnormalities were focal in five patients, multifocal in three patients, generalized in one patient, and mixed (focal/multifocal and generalized) in three patients. Findings were notable for a predominance of discharges over the posterior/occipital head regions in eight patients (66%) over 13 different studies in patients with diagnoses of MELAS (*n* = 6), POLG1 (*n* = 1), and MERRF (*n* = 1). In these patients, five patients experienced visual symptoms during seizures.

In the patients with normal or mildly abnormal outpatient EEG studies, four of the five patients continued to have normal or mildly abnormal outpatient EEG studies over time. Three patients with mild-to-moderately abnormal outpatient EEG studies had a clinical history of status epilepticus. Five patients with severely abnormal outpatient EEG recordings had a history of status epilepticus.

### Imaging Findings

**Figure 1:**
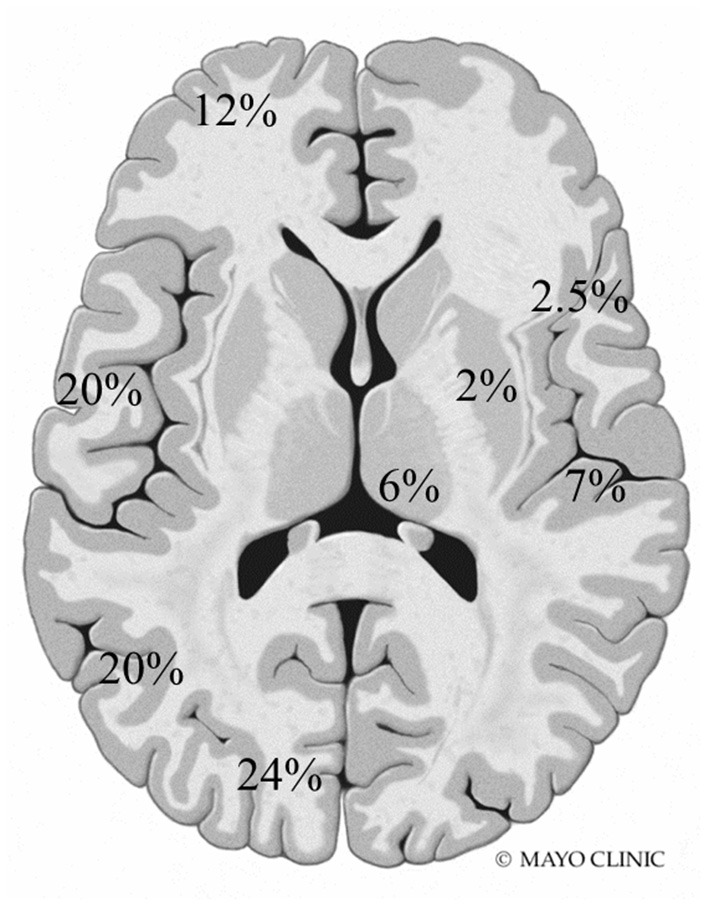
Frequency and location of MRI abnormalities. Right side: insula, perirolandic, basal ganglia, and thalamus. Left side: frontal, temporal, parietal, and occipital lobes (not shown: brainstem 0.5% and cerebellum 5%).

A total of 77 total MRI studies were performed. T2 hyperintensities were seen in 18 patients (90%) and abnormal diffusion restriction was present in 8 patients (40%). Imaging abnormalities were most commonly seen in the posterior head regions (Figure 1). Additional findings included cortical laminar necrosis (*n* = 3, 15%), global cerebral atrophy (*n* = 16, 80%), dysmyelination (*n* = 5, 25%), and abnormal contrast enhancement (*n* = 3, 15%).

**Figure 2:**
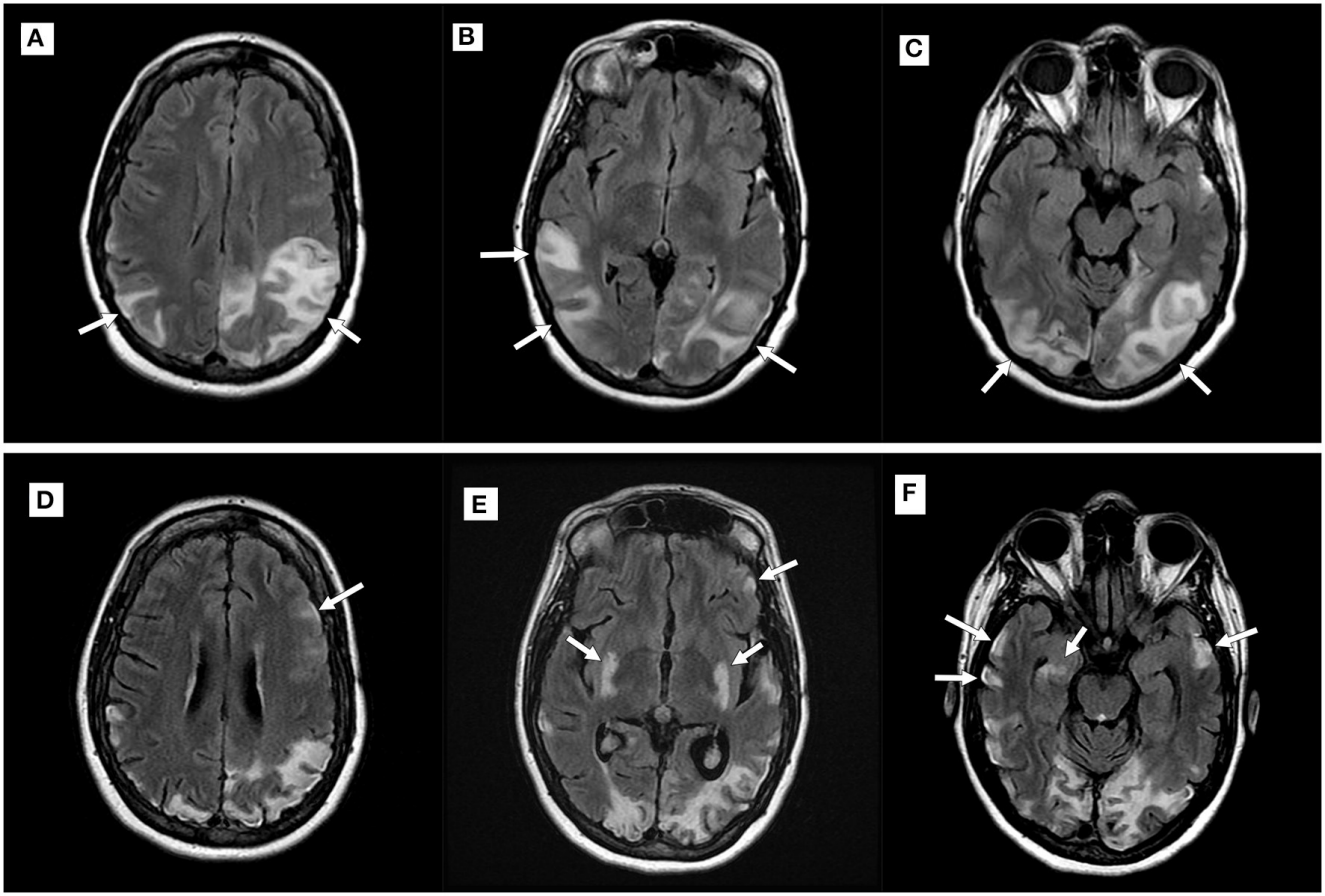
Imaging progression in a patient with mitochondrial encephalomyopathy, lactic acidosis, and stroke-like episodes (MELAS). Top row **(A–C)**: Axial T2 fluid-attenuated inversion recovery (FLAIR) imaging demonstrating new hyperintensities (white arrows) within the parietal and occipital lobes during an acute attack; Bottom row **(D–F)**: Imaging performed during a subsequent MELAS episode with new putaminal and bilateral temporal hyperintensities (white arrows). Note volume loss seen in previously affected areas.

Consecutive MRIs were performed in 17 patients and imaging evidence of disease progression was present in 11 patients [six patients with MELAS, two patients with POLG1, and the patients with MERRF, exosome component 3 (EXOSC3), and dynamin-like protein 1 (DNM1L)]. In the patients with MELAS, T2 hyperintensities and/or DWI abnormalities became more widespread over serial examinations, with subsequent abnormalities seen in the bilateral temporoparietal and parieto-occipital regions (Figure 2). In the two patients with POLG1-related disorders, the abnormalities spread to the temporoparietal regions over time. In the patient with MERRF, imaging abnormalities worsened but remained present over the frontal regions. In the patients with EXOCS3 and DNM1L, imaging abnormalities were seen in the parieto-occipital regions.

**Figure 3:**
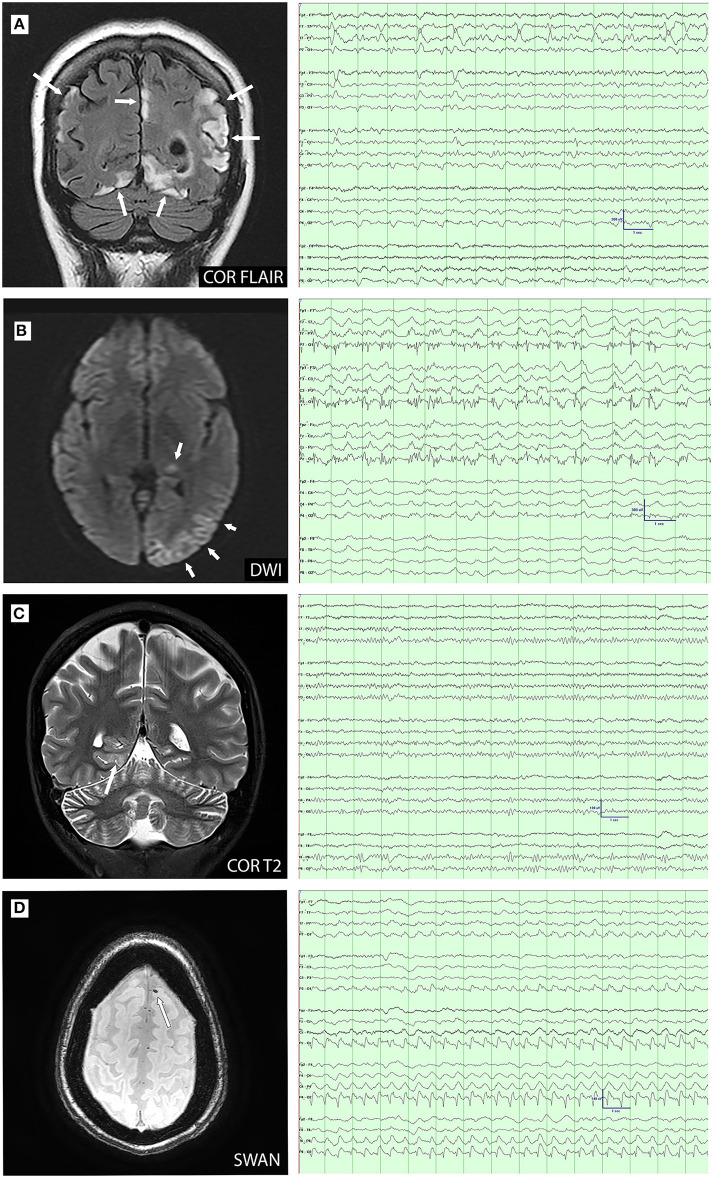
MRI-electroencephalogram (EEG) concordance **(A–C)** and discordance **(D)** comparisons. **(A)**: Coronal FLAIR image demonstrates cortical and subcortical hyperintensity in the parietal and occipital lobes. EEG demonstrates left temporal lateral periodic discharges and bioccipital spikes; **(B)**: Diffusion-weighted imaging (DWI) demonstrates restriction in the left pulvinar and temporo-occipital cortex. EEG shows ongoing electrographic seizure activity over the left posterior temporal and occipital; **(C)**: Coronal T2 image demonstrates cortical hyperintensity in the right medial occipitotemporal gyrus. EEG shows right posterior temporal slowing with increased right posterior region amplitudes; and **(D)**: Axial susceptibility-weighted angiography (SWAN) image demonstrates susceptibility artifact consistent with microhemorrhage in the left superior frontal gyrus. EEG demonstrates right posterior lateralized periodic discharges.

A total of 16 patients had MRI and EEG studies obtained within 30 days of each other (range 0–27 days, mean 7.22 days). A total of 24 MRI-EEG time points were available for review, of which, 13 time points (in nine patients) were concordant and 11 time points (in seven patients) were discordant (Figure 3). The 13 concordant time points corresponded to a period of new or worsening symptoms (i.e., new deficits, new stroke-like attack, breakthrough seizures, status epilepticus, etc.) with 10 of these studies (77%) performed in the acute inpatient setting. In nine of the discordant time points (82%), evaluations were performed in the outpatient setting during symptom/disease stability and the remaining two discordant time points occurred during acute episodes of focal motor status epilepticus.

### Antiseizure Medications

A total of 18 patients (90%) were initiated on ASM therapy during or prior to their first evaluation at our institution. The most commonly prescribed ASMs were levetiracetam (*n* = 13), phenytoin (*n* = 10), lamotrigine (*n* = 7), valproic acid (*n* = 6), and clonazepam (*n* = 3). One patient had a history of hyperammonemia and three patients had a history of transaminitis attributed to valproic acid. The patients on valproic acid were converted to another therapy once a diagnosis of a mitochondrial disorder was made. Side effects occurred in 11 patients (55%), most commonly reported as mood change or dizziness.

At last known follow-up, the median number of ASMs used were two per patient (range 0–5, SD 1.5). Of the medications at last follow-up, patients were often on levetiracetam (*n* = 9) and/or lamotrigine (*n* = 6). Five patients experienced at least 1 year of seizure freedom on monotherapy: three patients on lamotrigine, one patient with levetiracetam, and one patient with carbamazepine. One patient had significant reduction on oxcarbazepine to approximately three seizures per year. The remaining four patients on ASM and alive at last follow-up, continued to have refractory epilepsy despite use of one to five ASMs.

### Nutraceutical Supplementation (Mitochondrial Cocktail)

All patients were initiated on nutraceutical supplementation at the time of diagnosis and all patients remained on supplementation at last follow-up or time of death. The most commonly used supplements were carnitine (95%), coenzyme Q10 (90%), arginine (40%), and riboflavin (40%) with the number of supplements at initial evaluation ranging from one to nine (mean 3.9, SD 1.9). There was no significant difference seen between number of supplements used and clinical progression. In a subanalysis of 10 patients with MELAS, there was no statistically significant difference between the mean number of supplements and subsequent stroke-like episodes (*p* = 0.7). In adult patients who were still living at the end of the study period, there was no relationship seen between use of supplements and seizure control (*p* = 0.39), level of independence (*p* = 0.27), or mortality (*p* = 0.13).

### Functional Status and Follow-Up

The duration of follow-up ranged from 3 months to 31 years (mean 9 years). At last follow-up or patient contact, eight patients (40%) were deceased [five patients with MELAS (one patient with Ogilvie's syndrome/toxic megacolon, one patient with cancer, one patient with respiratory arrest, and two patients related to disease progression)], one patient with POLG (withdrawal of support in refractory myoclonic status epilepticus), one patient with Leigh syndrome (respiratory arrest), and one patient with DNM1L (withdrawal of support in refractory myoclonic status epilepticus). All the patients were assigned an initial and follow-up mRS score (Table 1). The mean initial mRS was 1 (range 0–4, SD 1.2), correlating to no significant disability, compared to last mRS mean of 4.65 (range 2–6, SD 1.4), corresponding to a moderately severe to severe disability and requiring assistance for all the needs. At last follow-up, no patients were mRS = 0.

## Discussion

This retrospective series of patients with mitochondrial disorders and epilepsy demonstrated an increasing predominance of posterior imaging and EEG abnormalities. Focal seizures, particularly with visual phenomena, were the most frequently seen, correlating with focal lesions commonly found in our cohort. As expected, patients with the same genetic variant had variable disease manifestations and clinical course.

On EEG studies, seizures and epileptiform abnormalities most commonly localized to the posterior temporal, parietal, and occipital regions. This proclivity for the posterior cerebral regions in mitochondrial disorders has been previously described and early recognition of such findings should suggest the possibility of a mitochondrial cytopathy (7, 15, 26).

Our findings are supported by previous patient series and literature reviews on epilepsy features in mitochondrial disorders (Table 3) (8, 10, 16, 28–31). Focal seizures were the predominant seizure type in our series, which is in agreement with the previous review by Anagnostou et al. in POLG and a recent case series in MELAS by Li et al. (10, 28). In a series of 19 patients with POLG1, occipital rhythmic slowing was present in 7 patients and occipital interictal discharges was present in 18 patients, demonstrating an occipital predilection in POLG (15). This finding, as seen in our study over the disease course, is concordant with electrophysiologic data reported by other authors (10, 16, 28, 29). Occipital status epilepticus and occipital seizures have also been previously reported in MELAS; this manifestation may precede other clinical signs of the disease and serve as a potential diagnostic clue (29, 32, 33). Similarly, in our series, MRI abnormalities frequently involved the posterior head regions, often in concordance with EEG findings, which is in agreement with other series (18, 34, 35).

**Table 3:** Review of selected studies on epilepsy in patients with mitochondrial disorders.

**References**	**Population (N), Study type**	**Disorder(s), genetic variants (n)**	**Seizure semiology, n (%)**	**EEG epileptiform abnormality locations and frequency**	**Imaging findings location and frequency**
Fine et al. (27)	pEDIATRIC and adult (20) Retrospective	Multiple:MELAS, - m.3243A>G (10) - m.13513G>A (1) - m.3251A>G (1)POLG1-c.1491G>C, c.2243G>C (3)MERRF, m.3256C>T (1)Leigh, m.13513G>A (1)PCH type 1B, c.395A>C (1)DNM1L, c.1207C>T (1)	Focal only 18 (90%) Focal to bilateral tonic-clonic 6 (30%) Focal and generalized 1 (5%) Myoclonic 4 (25%) Epileptic spasms 1 (5%) Status epilepticus 15 (75%) - Focal sensory status 7 (46.7%)	Occipital 40% Focal 55% Multifocal 15% Generalized 25% LPDs 45%	Occipital/posterior 44% Temporal 20% Frontal 12% Thalamus 6% Basal ganglia 2% Global atrophy 80%
Li et al. (28)	Pediatric and adult (34) Retrospective	MELAS, m.3243A>G (34)	Focal 20 (58.8%) Generalized 11 (32.3%) Unknown 3 (8.8%)	Focal 69.57% Occipital 69.57% Frontal 65.22% Temporal 47.83% Parietal 34.78%	Occipital 75% Temporal 85.71% Parietal 85.71% Atrophy 64.29%
Lee et al. (8)	Pediatric (22) Retrospective	MELAS, m.3243A>G (22)	Focal only (50%), Focal and generalized (13.6%) Generalized only (1%) Status epilepticus (100%)	Focal 36.4% Multifocal 31.2% Generalized 27.3% LPDs 31.8%	Infarction 90.9% (location not specified) Basal ganglia 50% Thalamus 8% White matter 45.5% Atrophy 77.3%
Demarest et al. (29)	Pediatric (7) Retrospective	MELAS, m.3243A>G (7)	Focal 5 (71%) Status epilepticus 4 (57%) - Occipital status epilepticus 2 (50%)	Focal 57% Generalized 14% Occipital/posterior 37.5% Frontal 12.5% LPDs 12.5%	N/A
Specchio et al. (16)	Pediatric and adult (195) Meta-analysis/Review	POLG - c.1399G>A-c.2243G>C-c.2542G>A(74.2% of variants identified)	*N* = 183 Myoclonic 95 (52%) Focal motor 62 (34%) Focal to bilateral tonic-clonic 81 (44%) Status epilepticus 85 (46%)	*N* = 77 Occipital 19.5% Centroparietal 14% Diffuse 6.5% LPDs 1%	*N* = 109 Occipital 38% Temporal/parietal 15.5% Frontal 9% Thalamus 40% Basal ganglia 5.5% Atrophy 26.6%
Anagnostou et al. (10)	Pediatric and adult (372) Meta-analysis/Review	POLG - c.1399G>A (215) - c.2243G>C (70) - c.2542G>A (26)	*N* = 229 Focal motor 146 (64%) Myoclonic 133 (58%) Status epilepticus - Generalized status 112 (49%) - Focal motor status 78 (34%)	*N* = 72Occipital/posterior 61%Frontal 6%Temporal 2%Multifocal 23%	*N* = 136 Stroke-like lesion 43% -Occipital 86% -Parietal 20% -Frontal 7% -Temporal 1.5% Thalamus 37% Basal ganglia 14% Atrophy 28%
Chevallier et al. (30)	Pediatric and adult (165) Retrospective	Multiple (detailed genetic variants not available):Leigh (16)MELAS (9)POLG/SANDO (2)MERRFDNA depletion syndrome (5)	*N* = 60 Focal 43% Generalized tonic-clonic 37% Myoclonic 22% Infantile spasms 7%	*N* = 109 Focal 39% Generalized 39%, Multifocal 41% Generalized and multifocal 25%	N/A
Lorenzoni et al. (31)	Adult (6) Retrospective	MERRF-m.8344A>G (5)	Myoclonic 6 (100%) Generalized tonic-clonic 6 (100%)	Generalized 83%	Diffuse atrophy 66%

*DNM1L, dynamin-like protein 1; LPDs, lateralized periodic discharges; MELAS, mitochondrial encephalomyopathy, lactic acidosis, and stroke-like episodes; MERFF: myoclonic epilepsy with ragged-red fibers; PCH, pontocerebellar hypoplasia; POLG, polymerase gamma; SANDO, sensory ataxia, neuropathy, dysarthria, and ophthalmoparesis*.

The most common mitochondrial disorder in our cohort was MELAS. Clinically, this diagnosis should be considered in an individual, particularly a young individual, with recurrent stroke-like episodes of unclear etiology. Stroke in MELAS is hypothesized to result from a combination of impaired oxidative phosphorylation, abnormal nitric oxide homeostasis, and vascular endothelial dysfunction (36–38). Oxidative stress leads to abnormal calcium homeostasis producing neuronal hyperexcitability (39, 40). The resultant localized cortical dysfunction and cerebral edema in acute and recovering cerebral infarcts increases the risk for seizure. Seizures then lead to increased free radical production within dysfunctional mitochondria, predisposing to further oxidative damage (5). With prolonged seizures, this process may lead to cortical laminar necrosis, which is manifested in the acute phase as T2 hyperintense or DWI hyperperfusion signal in the cortical ribbon on MRI (37, 41). The combination of local cortical injury and ongoing seizures then could produce a structural lesion (i.e., encephalomalacia, gliosis, etc.) predisposing to future seizure activity.

Inexorable progression was seen in our patients, with recurrent seizures, stroke-like episodes, and further brain injury, leading to continued functional decline. The majority developed moderate-to-severe disability or death, with eight patients deceased, of which seven deaths were attributable to their underlying mitochondrial disorder.

The treatment of seizures in patients with mitochondrial disorders is challenging. There is known mitochondrial toxicity from several ASMs, notably, valproic acid, phenytoin, and phenobarbital (42, 43). In our series, it was rare for patients to not have tried two or more ASMs including valproic acid, phenytoin, and phenobarbital. Interestingly, two patients were not started on ASM while seen at our institution, despite recurrent seizures. The reason documented for both was patient/family preference. The clinical course of the epilepsy in this cohort was unfortunately dismal, with the majority experiencing intractability and two of whom died from complications of super-refractory status epilepticus.

We found no evidence of efficacy of nutraceutical supplementation in our cohort, with the majority experiencing significant progression and exacerbations despite treatment. Compliance with the mitochondrial cocktail was difficult to assess based on chart review alone, which limited our ability to be certain as to the futility of therapy. These regimens can be difficult to maintain and are cost prohibitive. Some authors report the benefits of arginine and citrulline during acute attacks in MELAS to mitigate cortical injury by free radical reduction (36, 44, 45). Indeed, our practice is to include arginine in the regimen of patients with MELAS and provide additional supplementation during a stroke-like episode. However, the results of this retrospective review are sobering as to the potential for response to nutritional therapies in these patients. The use of other supplements has not been shown to alter disease course in mitochondrial disorders (45). A prospective clinical trial would be necessary to definitively evaluate the efficacy of supplement use in mitochondrial disorders.

There are several limitations of this study. As this study was retrospective, we were limited to information contained within the medical record. A large number of patients were excluded due to missing or incomplete records. We only included those with genetically confirmed disease, thus further reducing our sample size and potentially limiting our cohort to the most severely affected. Our small series was heavily weighted toward patients with MELAS, which could have skewed our findings and interpretation.

## Conclusion

This retrospective series reviewed the clinical course of 20 patients with mitochondrial disorders and epilepsy. Most patients had EEG and neuroimaging abnormalities over the posterior head regions or showed progression to involvement of these regions over time. Seizures with visual symptoms occurred frequently. The presence of posterior predominant EEG and MRI abnormalities of unclear etiology should increase clinical suspicion of a mitochondrial disorder, particularly in an adult patient presenting with occipital status epilepticus. Our results raise question as to the efficacy of nutraceutical therapy for these diseases. A larger prospective study would be helpful to further clarify the optimal medical management of patients with mitochondrial disorders and epilepsy.

## Data Availability Statement

The original contributions presented in the study are included in the article/supplementary material, further inquiries can be directed to the corresponding author/s.

## Ethics Statement

The studies involving human participants were reviewed and approved by Mayo Clinic IRB. Written informed consent from the participants' legal guardian/next of kin was not required to participate in this study in accordance with the national legislation and the institutional requirements.

## Author Contributions

AF wrote the first draft of the manuscript, collected, analyzed and interpreted data, and revised the manuscript. GL collected and interpreted data and revised the manuscript. RG and JB revised the manuscript and gave final approval. All the authors contributed to the article and approved the submitted version.

## Conflict of Interest

The authors declare that the research was conducted in the absence of any commercial or financial relationships that could be construed as a potential conflict of interest.

## Publisher's Note

All claims expressed in this article are solely those of the authors and do not necessarily represent those of their affiliated organizations, or those of the publisher, the editors and the reviewers. Any product that may be evaluated in this article, or claim that may be made by its manufacturer, is not guaranteed or endorsed by the publisher.
